# Convenience Store Use and the Health of Urban Adolescents in Seoul, South Korea

**DOI:** 10.3390/ijerph17186486

**Published:** 2020-09-06

**Authors:** Nan-He Yoon, Changwoo Shon

**Affiliations:** 1 Department of Health Administration, Hanyang Cyber University, Seoul 04763, Korea; nhyoon@hycu.ac.kr; 2 Department of Urban Society Research, The Seoul Institute, Seoul 06756, Korea

**Keywords:** adolescents, convenience store, self-rated health, nutrition, urban area

## Abstract

To improve urban adolescents’ dietary behaviors and health, factors that influence them to buy meals in convenience stores with regard to urban food environments must be determined. This study investigated the factors which influence adolescents’ substitution of meals with convenience store meals and its impact on their health in Seoul (South Korea). Multilevel analysis and logistic regression analysis were conducted using data from the Korea Youth Risk Behavior Survey, a national health survey with a representative sample of Korean adolescents. Among 17,624 teenagers who responded to surveys in 2017 and 2019, 30.5% of them substituted meals with convenience store meals more than three times a week. Girls and students with a lower family economic level were more likely to frequently consume food from convenience stores. Unhealthy lifestyles and poor mental health status also influenced their decisions to substitute meals with convenience store food. Those who frequently consumed meals from convenience stores were more likely to have unhealthy lifestyles, featuring bad diets, smoking, drinking, and sedentary behaviors. They also reported significantly poorer self-rated health and mental health. To promote healthy lifestyles among adolescents, efforts to raise awareness and develop supportive environments for healthy diets are strongly recommended.

## 1. Introduction

Adolescence is a very important period for physical and emotional growth and development, and adolescents are an important population group in terms of health promotion because most health behaviors are formed at this time. The habituation of health behaviors including dietary behaviors is the most critical factor for the prevention of chronic diseases, which are a major health problem of late, and is also important for strengthening immunity to prevent infectious diseases [1,2,3].

Meanwhile, adolescents’ health is more likely to be affected by the socioecological environment and context around them than it is for adults. In particular, the built environment inside and outside of schools, where adolescents spend most of their time, and peer influence have a significant effect on their practice of healthy behaviors [4,5].

There are 24 h convenience stores in many countries, but their locations and functions are different. In Western countries, convenience stores are usually located next to gas stations and tend to provide light snacks and drinks to drivers. But in Korea and Japan, they are often located in urban areas with floating populations and offer a much wider variety of products than in the West. Therefore, urban Korean residents have particularly high access to convenience stores and often seek meals there. In particular, Korean adolescents, who have a culture of studying through private academies rather than schools until late at night, can be said to have a stronger tendency to frequent these stores [6].

Meanwhile, the problem of unhealthy diets and nutritional imbalances among Korean adolescents has been taken very seriously. Due to their heavy academic burden and lack of free time, it is common for Korean adolescents to have irregular meals and substitute meals with instant food or convenience food bought at fast food outlets or convenience stores [7,8,9]. Thus, since 2017, the Korea Youth Risk Behavior Survey (KYRBS), which is a national health survey with a representative sample of Korean adolescents, has contained items about their dietary behaviors at convenience stores. In the survey results, the proportion of adolescents who substituted meals with any foods from convenience stores at least once a week rose from 65.3% in 2017 to 69.1% in 2019. Those who had meals at convenience stores more than three times a week increased from 26.0% in 2017 to 29.3% in 2019. Additionally, as of 2019, 70.1% and 32.3% of adolescents in Seoul reported having meals at convenience stores more than once a week and three times a week, respectively, which is higher than the national average. Compared to this, in an analysis of convenience store use by U.S. adolescents, 47.5% of respondents visited convenience stores weekly [10]. It was found that Korean adolescents use convenience stores more often than in other countries, especially in Seoul. Therefore, it is urgent for adolescents in urban areas in Korea to make efforts to promote their health through healthy eating and a good nutritional balance, as it is common to eat at convenience stores.

Convenience foods are generally high in calories, fat, sugar, or salt and deficient in nutrients, which negatively affects the health of adolescents [11,12]. In addition to unhealthy dietary behaviors, those with higher accessibility to convenience stores or who visit them more often also exhibited a higher probability of smoking or drinking [13,14]. As such, the unhealthy diets of adolescents due to convenience store visits have a significant effect on obesity and are also significantly related to mental health and self-rated health [15,16,17]. Simultaneously, adolescents with unhealthy lifestyles and risky behaviors were found to visit convenience stores more often, which could lead to a vicious cycle of negative health effects and risks [10,18,19].

To improve urban adolescents’ dietary behaviors and health, it is necessary to understand the current status and context. In particular, the accessibility of convenience stores for urban residents is highly likely to affect their eating habits, especially for adolescents. As mentioned earlier, Asian and Western convenience store cultures are somewhat different. This study focused on youth in Seoul, South Korea, which is representative of Asian countries with their high convenience store utilization rates. Unfortunately, there have been few studies on adolescents’ use of convenience stores and health from an academic perspective. Although some research was conducted to elicit the factors affecting adolescents’ convenience store use [8,20,21], it did not take into account the environmental factors around schools and the relationship between meals at convenience stores and health.

Therefore, we investigated the factors influencing the experience and the impact of frequent meals at convenience stores of adolescents in Seoul using survey data on a representative sample. This study aimed to provide empirical evidence for policy proposals for improving the dietary lifestyles of urban adolescents by identifying the current status of convenience store use and its impact on health.

## 2. Materials and Methods

### 2.1. Data Source and Study Population

This study utilized data from the KYRBS, which is an annual survey that seeks to understand the current status of the health behaviors of adolescents in Korea and to present health indicators required for planning and evaluating health promotion programs for adolescents. Since 2005, the Ministry of Health and Welfare and the Korea Centers for Disease Control and Prevention have conducted the survey yearly on about 60,000 students from 400 middle schools and 400 high schools across the country, selected using a stratified cluster sampling method. The survey was validated and consists of questionnaires about health status and health behaviors, including potential issues that affect adolescents such as smoking, drinking, physical activities, dietary lifestyles, obesity and weight control, mental health, sexual behavior, Internet and mobile use, drug use, and violence.

The study population included 17,624 students in Seoul who responded to the 2017 and 2019 KYRBS. Of the total respondents, 8751 (49.7%) boys and 8873 (50.3%) girls were included, and 8237 (46.7%) students were in middle school and 9387 (53.3%) students were in high school. The distribution of convenience stores, a key factor of this study, varies across regions; they are the most concentrated in the metropolitan Seoul area. Therefore, we limited the study’s scope to school-age adolescents receiving full-time education from schools in the metropolitan Seoul area to deal with schooling and health in urban environments. In addition to individual data from the KYRBS, public administrative statistics data at the community level was used to consider environmental factors including the distribution of convenience stores. The study received approval from the Institutional Review Board at Hanyang Cyber University (IRB No. 2018-005).

### 2.2. Outcome Variables

This study analyzed adolescents’ experiences of eating meals at convenience stores. The first outcome variable for the analysis was whether the respondents answered the question “How often have you substituted your meals with food from convenience stores or snack bars in the last seven days?” with “More than or equal to three days a week.”. The substitution of meals refers to consuming quick meals from convenience stores rather than homemade or school-provided meals using fresh ingredients. For the second analysis, the outcome variable was good self-rated health by those who frequently eat at convenience stores, defined as rating one’s health as “very healthy” or “healthy.”

### 2.3. Independent Variables

The analytic model included the socioeconomic characteristics of the respondents such as gender, grade, parents’ educational background, and residence patterns. Variables related to the respondents’ dietary behaviors, lifestyle, and mental health were included in the model to identify factors affecting the adolescents’ usage of convenience stores and health.

Dietary behaviors consisted of vegetable, fruit, fast food, soda, highly caffeinated beverage, and sugar-sweetened beverage intake. The questions asked about dietary behaviors included the frequencies of consuming such foods or drinks in a week. If the respondents replied that they consume fast food, soda, highly caffeinated beverages, or sugar-sweetened beverages more than three times a week, we categorized them as having an unhealthy diet. Meanwhile, in terms of vegetable and fruit intake, we defined those who had these at least once a day as having a healthy diet [22].

Other health behaviors including alcohol consumption, smoking, sedentary behaviors, and sleep duration were also considered. The frequencies of their alcohol consumption and smoking per month and per day, respectively, were asked in the survey to categorize them as current drinkers or smokers. There was a question regarding the duration of time spent sitting or lying down on weekends except when studying, working, or sleeping, and this was used in the analysis to specify if they had a sedentary lifestyle. About half of the respondents responded that they spend more than four hours engaging in sedentary behavior, so this category was divided into those who spend more than four hours and those who spend less than four hours being sedentary. As for the adolescents’ quality of daily life, the analysis included whether or not they perceived that they get enough sleep.

The mental health status of adolescents was measured based on stress perception and depression. When asked “How much stress do you usually feel?,” those who replied that they often or occasionally felt stressed were categorized as ‘often,’ and as ‘seldom’ if they replied that they seldom or never felt stressed. In terms of depression, they were categorized according to whether they had experienced being sad or depressed enough to the point that they could not continue with their daily lives for two weeks within the previous year.

Additionally, the number of convenience stores per 1000 people in the community where the respondents resided, which is a proxy variable for access to convenience stores, was also defined as an environmental factor. The number of convenience stores was collected from the census on establishments reported by Statistics Korea and the number of residents was collected from administrative data reported by the Korean Ministry of the Interior and Safety. Afterwards, the number of convenience stores was divided by the number of residents in each community and multiplied by 1000.

### 2.4. Data Analysis

The study population was first classified into those who ate meals at convenience stores more than or equal to three days a week and those who did not, and characteristics were compared between the two groups with chi-square tests.

Second, a multilevel logistic regression analysis was conducted on factors influencing the experience of eating at convenience stores more than three days a week. Individual factors including demographic and socioeconomic characteristics, dietary behaviors, lifestyle, and mental health status were analyzed as variables for level 1, and the distribution of convenience stores in the region was included in the model for level 2.

Finally, a multivariate logistic regression analysis was conducted to investigate the effects of adolescents’ frequent consumption of meals from convenience stores on their health status after adjusting for other individual factors. Control variables such as socio-demographic characteristics, lifestyles, and mental health status were also included in the model.

We tested for the significance of all analytic models referring to a significance level of 0.05. The statistical analyses were all conducted using the SAS ver. 9.4 program (SAS Institute INC, Cary, NC, USA).

## 3. Results

### 3.1. General Characteristics of the Study Population

Of the total 17,624 respondents, 5371 (30.5%) replied that they substituted their meals with food from convenience stores more than or equal to three days a week (Table 1). There was a statistically significant association between the frequency of consuming food from convenience stores and the respondents’ general characteristics.

In total, 27.9% of boys and 28.2% of middle school students said they ate meals at convenience stores more than or equal to three days a week, while 33.1% of girls and 32.5% of high school students did (*p* < 0.001). Proportions of adolescents to have meals more than three times a week with higher parental educational levels or higher economic household status were found to be lower (*p* < 0.001). Furthermore, adolescents who often experienced heavy stress or depression were more likely to have their meals at convenience stores frequently (*p* < 0.001).

Significant differences were also identified in students’ dietary behaviors and lifestyles (Table 2). Regarding dietary behaviors, those who ate vegetables or fruits more than once a day had a lower percentage of frequent meals at convenience stores (*p* < 0.001). Adolescents with unhealthy dietary behaviors such as consuming fast food, soda, highly caffeinated beverages, and sugar-sweetened beverages more than three times a week had a significantly higher frequency of meals at convenience stores (*p* < 0.001).

In terms of an unhealthy lifestyle, 42.3% of those who drank alcohol more than once a month and 48.6% of those who smoked more than one cigarette a day responded that they ate meals at convenience stores more than three times a week, with a significant increase compared to other respondents (*p* < 0.001). It was also found that adolescents with more than four hours of sedentary time on weekends or those who did not have sufficient sleep duration had meals at convenience stores more often (*p* < 0.001).

### 3.2. Factors Affecting Experiences of Substituting Meals with Foods from Convenience Stores

Table 3 shows how much each factor affects the frequency of substituting meals for convenience store food. Girls (*p* = 0.003) were more likely to eat meals at convenience stores than boys. The higher their family’s economic level (*p* = 0.010), the less likely they were to eat at convenience stores more than or equal to three times a week.

The distribution of convenience stores in the region did not significantly affect adolescents’ experiences of eating at them (*p* = 0.371). However, after controlling for access to convenience stores in the region, the dietary behaviors, lifestyles, and mental health factors of adolescents were found to have a significant impact on eating frequent meals at convenience stores.

Adolescents who ate vegetables (*p* = 0.017) or fruits (*p* = 0.013) at least once a day were less likely to have meals frequently at convenience stores compared to those who did not. However, respondents who frequently consumed fast food (*p* < 0.001), soda (*p* = 0.002), highly caffeinated drinks (*p* = 0.002), or sugar-sweetened beverages (*p* = 0.001) were more likely to have their meals at convenience stores more than three times a week. The likelihood of eating meals at convenience stores more than or equal to three times a week was higher for those who drank alcohol more than once a month (*p* = 0.014) or smoked more than one cigarette a day (*p* = 0.034) compared to non-drinkers and non-smokers.

Furthermore, adolescents who perceived that they did not get enough sleep were more likely to eat at convenience stores frequently than those who got enough sleep (*p* = 0.007). In terms of mental health, respondents who felt more stressed (*p* = 0.008) or depressed (*p* = 0.006) were more likely to have more frequent meals at convenience stores.

### 3.3. Effects of Experiences Eating at Convenience Stores on Self-Rated Health

Finally, the results of the logistic regression analysis conducted to investigate the impact of frequent meals at convenience stores on the self-rated health of adolescents are shown in Table 4. The results for most variables showed significant effects in the opposite direction with the factors influencing frequent meals at convenience stores.

Girls (OR = 0.708; 95% CI 0.659–0.762) and high school students (OR = 0.740; 95% CI 0.689–0.795) reported poorer self-rated health than boys or middle school students, respectively. Students with higher family economic status (OR = 1.716; 95% CI 1.542–1.910, OR = 1.273; 95% CI 1.150–1.409) perceived their health as good.

Adolescents who ate vegetables (OR = 1.303; 95% CI 1.211–1.402) or fruits (OR = 1.177; 95% CI 1.080–1.283) every day evaluated their own health more highly than those who did not. Those who drank soda (OR = 0.904; 95% CI 0.835–0.979) or highly caffeinated beverages (OR = 0.852; 95% CI 0.766–0.947) more than three times a week were less likely to have good self-rated health compared to those who did not. Meanwhile, respondents who had more than four sedentary hours on weekends (OR = 0.865; 95% CI 0.807–0.927) or who had an insufficient sleep duration (OR = 0.643; 95% CI 0.584–0.708) perceived their health as relatively poor. Those who faced severe stress (OR = 0.445; 95% CI 0.414–0.479) or depression (OR = 0.706; 95% CI 0.653–0.762) had poor self-rated health.

Further, after controlling the students’ individual factors affecting self-rated health, including lifestyle and mental health, it was found that the self-rated health of adolescents who had their meals at convenience stores more than or equal to three times a week was relatively poor compared to those who did not (OR = 0.810; 95% CI 0.750–0.875).

## 4. Discussion

This study investigated factors influencing and resulting from Korean adolescents’ eating experiences at convenience stores. The results of the analysis showed that students with unhealthy dietary behaviors, a lot of sedentary time, a lack of sleep, more severe stress, and depression were more likely to have their meals at convenience stores more frequently. This correlation was significant even after controlling the accessibility of convenience stores, which was measured based on the distribution of convenience stores in the region. Furthermore, adolescents who frequently ate at convenience stores had relatively poor self-rated health.

Several studies have been conducted recently on adolescents’ use of convenience stores. Convenience stores are one of the most accessible retail environments for urban adolescents to purchase various products including food and beverages [23,24,25]. In contrast to grocery stores or supermarkets, options for healthy food and beverages are limited in convenience stores, and unhealthy foods dominate available stock [12,26,27,28,29]. Furthermore, because of advertising and marketing, many adolescents are exposed to unhealthy diets. According to Horsley, who examined the types of food children were exposed to at the checkout counters of convenience stores, 89% of stores had unhealthy items on display [30]. This dietary environment also promotes unhealthy eating among adolescents by increasing their exposure to unhealthy foods [31]. According to prior studies, adolescents who frequently visit convenience stores are more likely to drink unhealthy beverages such as soda and sugar-sweetened drinks [32], and less likely to consume healthy foods such as vegetables and fruits [33]. Therefore, efforts have been made to secure a healthier environment for adolescents.

Consistent with previous studies on adolescents’ convenience store use and health [10,34], the unhealthy or risky behaviors that had significant negative effects on this population’s health were found to promote frequent eating at convenience stores for adolescents in Seoul.

According to the results of the descriptive analysis, adolescents who consumed more fast food, soda, or highly caffeinated beverages tended to substitute their meals for convenience store food more frequently. However, the opposite was found for those who ate vegetables and fruits daily. Choosing foods based on taste or convenience—resulting in quick and easy eating—might have resulted from personal preference rather than unhealthy dietary habits [15,27,35,36]. Such eating behaviors may cause not only negative effects on their health due to the nutritional imbalances, but these can also expose them to risk factors that become lifestyle habits leading to chronic diseases, and even lead to poorer school performance [37].

It is also noteworthy that adolescents who indulged in risky behaviors such as smoking and drinking ate more meals at convenience stores. Smoking and drinking by teenagers is not permitted in Korea. Nevertheless, 4.5% of respondents said they smoked more than one cigarette every day, and 13.7% reported drinking alcohol more than once a month. In fact, according to the results of the KYRBS in 2017, 48.0% of smokers and 32.1% of drinkers chose convenience stores as places to buy alcohol or cigarettes. Adolescents living in areas with higher access to convenience stores are more likely to drink or smoke [10,14,38]. This shows that a convenience store is an environment where teenagers can be easily exposed to health risks.

Another notable result is that girls substituted their meals with convenience food more frequently compared to boys. Excessive weight control and being underweight are severe health problems faced by Korean female teenagers. There is a high probability for girls to eat small amounts of meal replacement foods which are unbalanced and relatively deficient in nutrition. These can lead to other health problems. Therefore, the context and characteristics of convenience stores need to be considered to suggest healthier food options.

However, unhealthy or risky behaviors among adolescents are a complicated problem that cannot be expected to be changed quickly or simply. Most Korean students are under severe stress due to excessive academic burdens. It is common for Korean adolescents to study at school from early morning to late afternoon and continue to take classes or study at another academy until late at night. This pattern continues on weekends. The priority of students, parents, and schools during students’ adolescence is academic performance more than practicing healthy behaviors. Therefore, they tend to overlook health promotion and exposure to unhealthy environments. This is especially true for dietary behaviors, where immediate health outcomes are not easily noticeable.

In this sense, among the factors included in the analysis of this study, sedentary time, daily sleep duration, and the perception of stress or depression are important to discuss. In the results of the empirical analysis, respondents with long sedentary periods or poor sleep duration often substituted their meals at convenience stores, and they evaluated their own health as poor. In adolescence, regular physical activity and sufficient sleep are very important not only for health promotion but also for normal development. However, only 22.0% of adolescents said they got enough sleep, while 78.0% said they lacked sufficient rest.

As such, those who spend less time under perceived control are more likely to be driven to choose simple, high-calorie and highly salty, spicy, or sweet foods rather than healthy options. They are also relatively vulnerable to mental health problems. According to the results of the analysis, the respondents who faced severe stress or depression on a daily basis often experienced unhealthy diets and evaluated their own health negatively. Unfortunately, the most frequent cause of death for teenagers in Korea is suicide, accounting for approximately 35.7% of total deaths, and it is increasing [39]. Mental health problems are usually significantly related to their health behaviors and lifestyles, including their diet, which also affect their health status. Therefore, more efforts are needed to protect and promote adolescents’ mental health, and the practice of health behaviors is also required to promote their mental health.

Based on our analysis of the KYRBS data, the most common meal substitutes that adolescents chose were instant noodles, rice balls, beverages, and snacks, most of which are lacking in nutrients and contain a high proportion of salt, fat, or sugar. Additionally, many teenagers gave “It is convenient to eat” (26.5%) as the reason why they ate at convenience stores, followed by “I don’t have time” (19.8%). This means that adolescents were forced to substitute their meals at convenience stores due to the social environment. It is clear that such foods as a substitute for a proper meal are far from healthy.

Convenience store use itself should not be considered unhealthy. It provides social, cultural, economic, and technical support for urban residents, who often seek time-saving alternatives in their busy daily lives [40]. With the increase in convenience store use in urban areas and the emphasis on the role of small retailers selling daily necessities in the region, the types of food sold by convenience stores are becoming more diverse, and the promotion of nutritious snacks is also increasing. Tran measured and compared calories in U.S. convenience store foods from 2013 to 2017 and concluded that the average calories decreased over five years [17]. This can be interpreted as a comprehensive result of a federal system mandating the labeling of nutritional information and the increasing demand for healthy foods among the population.

Efforts to create a supportive environment and system are essential to enable adolescents to achieve their health goals, including healthy eating, and encourage them to practice healthy behavior. Retail stores including convenience stores should ensure the availability of healthy options containing various nutrients. Convenience stores should also refrain from advertising and minimize the exposure of unhealthy foods, alcohol, and cigarettes that threaten health. Additionally, health education should be mandatory and practical opportunities should be provided so that healthy and nutritious alternatives can be selected among various options. It is also necessary to improve social awareness to encourage adolescents to consider their health as a priority so that they can enjoy healthy and fresh meals at designated places such as schools and homes.

The data analyzed in this study were cross-sectional data, which were limited in explaining the causal relationship between the factors. However, the findings can be used as a basis for policy proposals, as this study attempted to confirm a generalizable association by conducting empirical analyses using a representative sample. To prevent chronic diseases, it is important to habituate normal health-related behaviors. In particular, adolescence is a time when one’s lifestyle is determined, and it is necessary for everyone to recognize that risk factors during this period can accumulate as personal health threats, making it more important to seek improvements and pursue collaborative efforts.

This study was conducted on youth in Seoul, the capital city of Korea, but the findings have great implications for the eating habits and health of Asian adolescents who share a similar culture. Furthermore, it is expected that the study will also have major implications for Western adolescents, who often visit fast food stores or food trucks.

## 5. Conclusions

Using the KYRBS, a representative survey of Korean adolescents, this study investigated the influencing factors and health results of eating experiences at convenience stores for adolescents in Seoul. According to the results, adolescents who frequently substituted meals for food bought at convenience stores had unhealthy dietary habits and often exhibited risky behaviors such as smoking, drinking, or being sedentary. Furthermore, their unhealthy eating lifestyle made them vulnerable to physical and mental health problems. Although adolescence is a critical period for the establishment of health attitudes and lifestyles which determine one’s exposure to health risk factors, health promotion has often been overlooked due to adolescents prioritizing their academic performance. Efforts to develop a supportive urban environment for healthy dietary practices and encourage teens to make healthy decisions are strongly recommended.

## Figures and Tables

**Table 1:** Differences in Frequencies of Substituting Meals for Convenience Store Food According to Socio-Demographic Characteristics and Health Status of the Study Population.

Frequencies of Substituting Their Meal at Convenience Stores		<3 Times a Week		≥3 Times a Week		Total		χ^2^ (*p*-Value)
Variables		*n*	(%)	*n*	(%)	*n*	(%)	
**Gender**	Boys	6313	(72.1)	2438	(27.9)	8751	(49.7)	56.133 (<0.001)
	Girls	5940	(66.9)	2933	(33.1)	8873	(50.3)	
**School**	Middle school	5918	(71.8)	2319	(28.2)	8237	(46.7)	39.355 (<0.001)
	High school	6335	(67.5)	3052	(32.5)	9387	(53.3)	
**Father’s education**	≤High school	5701	(67.1)	2799	(32.9)	8500	(48.2)	46.663 (<0.001)
	≥College	6552	(71.8)	2572	(28.2)	9124	(51.8)	
**Mother’s education**	≤High school	6049	(66.9)	2990	(33.1)	9039	(51.3)	59.357 (<0.001)
	≥College	6204	(72.3)	2381	(27.7)	8585	(48.7)	
**Economic** **Status**	Upper	5598	(72.5)	2122	(27.5)	7720	(43.8)	78.874 (<0.001)
	Middle	5150	(68.4)	2381	(31.6)	7531	(42.7)	
	Lower	1505	(63.4)	868	(36.6)	2373	(13.5)	
**Living with family members**	Yes	11,979	(69.7)	5219	(30.3)	17,198	(97.8)	2.519 (0.113)
	No	253	(65.9)	131	(34.1)	384	(2.2)	
**Stress perception**	Seldom	7764	(74.1)	2708	(25.9)	10,472	(59.4)	259.513 (<0.001)
	Often	489	(62.8)	2663	(37.2)	7152	(40.6)	
**Depression**	Not experienced	9180	(73.3)	3342	(26.7)	12,522	(71.1)	292.696 (<0.001)
	Experienced	3073	(60.2)	2029	(39.8)	5102	(28.9)	
**Self-rated health**	Poor	3234	(61.9)	1991	(38.1)	5225	(29.6)	204.051 (<0.001)
	Good	9019	(72.7)	3380	(27.3)	12,399	(70.4)	
**Total**		12,253	(69.5)	5371	(30.5)	17,624	(100.0)	

**Table 2:** Differences in Frequencies of Substituting Meals for Convenience Store Food According to Lifestyles of Study Population.

Frequencies of Substituting Their Meal at Convenience Stores		<3 Times a Week		≥3 Times a Week		Total		χ^2^ (*p*-Value)
Variables		*n*	(%)	*n*	(%)	*n*	(%)	
**Vegetable intake**	<1 time a day	7075	(66.8)	3520	(33.2)	10,595	(60.1)	94.661 (<0.001)
	≥1 time a day	5178	(73.7)	1851	(26.3)	7029	(39.9)	
**Fruits intake**	<1 time a day	9049	(67.8)	4305	(32.2)	13,354	(75.8)	80.767 (<0.001)
	≥1 time a day	3204	(75.0)	1066	(25.0)	4270	(24.2)	
**Fast food intake**	<3 times a week	10,221	(75.6)	3299	(24.4)	13,520	(76.7)	1011.162 (<0.001)
	≥3 times a week	2032	(49.5)	2072	(50.5)	4104	(23.3)	
**Soda drinking**	<3 times a week	8709	(75.5)	2825	(24.5)	11,534	(65.4)	563.859 (<0.001)
	≥3 times a week	3544	(58.2)	2546	(41.8)	6090	(34.6)	
**Highly caffeinated beverage drinking**	<3 times a week	11,240	(72.0)	4362	(28.0)	15,602	(88.5)	406.786 (<0.001)
	≥3 times a week	1013	(50.1)	1009	(49.9)	2022	(11.5)	
**Sugar-sweetened beverage drinking**	<3 times a week	6736	(78.4)	1859	(21.6)	8595	(48.8)	619.695 (<0.001)
	≥3 times a week	5517	(61.1)	3512	(38.9)	9029	(51.2)	
**Alcohol drinking**	<1 time a month	10,856	(71.4)	4346	(28.6)	15,202	(86.3)	185.931 (<0.001)
	≥1 time a month	1397	(57.7)	1025	(42.3)	2422	(13.7)	
**Smoking**	<1 cigarette a day	11,841	(70.4)	4982	(29.6)	16,823	(95.5)	129.588 (<0.001)
	≥1 cigarette a day	412	(51.4)	389	(48.6)	801	(4.5)	
**Sedentary time at weekend leisure time**	<4 h	6321	(71.1)	2574	(28.9)	8895	(50.5)	20.047 (<0.001)
	≥4 h	5932	(68.0)	2797	(32.0)	8729	(49.5)	
**Sleep duration**	Not enough	9207	(67.0)	4532	(33.0)	13,739	(78.0)	185.455 (<0.001)
	Enough	3046	(78.4)	839	(21.6)	3885	(22.0)	
**Total**		12,253	(69.5)	5371	(30.5)	17,624	(100.0)	

**Table 3:** Results of Multilevel Analysis for the Factors Influencing Substitution of Meals with Convenience Store Food.

Independent Variables		b	S.E. ^1^	*p*
Individual Factors				
**Gender**	Boys	(ref.)		
	Girls	0.341	0.038	0.003
**School**	Middle school	(ref.)		
	High school	0.015	0.037	0.713
**Father’s education**	≤High school	(ref.)		
	≥College	−0.012	0.049	0.823
**Mother’s education**	≤High school	(ref.)		
	≥College	−0.132	0.049	0.074
**Economic Status**	Upper	−0.208	0.056	0.010
	Middle	−0.059	0.054	0.314
	Lower	(ref.)		
**Living with family members**	Yes	(ref.)		
	No	0.076	0.119	0.568
**Vegetable intake**	<1 time a day	(ref.)		
	≥1 time a day	−0.182	0.037	0.017
**Fruits intake**	<1 time a day	(ref.)		
	≥1 time a day	−0.233	0.044	0.013
**Fast food intake**	<3 times a week	(ref.)		
	≥3 times a week	0.853	0.040	<0.001
**Soda drinking**	<3 times a week	(ref.)		
	≥3 times a week	0.410	0.039	0.002
**Highly caffeinated beverage drinking**	<3 times a week	(ref.)		
	≥3 times a week	0.584	0.052	0.002
**Sugar-sweetened beverage drinking**	<3 times a week	(ref.)		
	≥3 times a week	0.533	0.038	0.001
**Alcohol drinking**	<1 time a month	(ref.)		
	≥1 time a month	0.270	0.053	0.014
**Smoking**	<1 cigarette a day	(ref.)		
	≥1 cigarette a day	0.319	0.086	0.034
**Sedentary time at weekend leisure time**	<4 h	(ref.)		
	≥4 h	0.059	0.035	0.194
**Sleep duration**	Not enough	0.320	0.047	0.007
	Enough	(ref.)		
**Stress perception**	Seldom	(ref.)		
	Often	0.243	0.038	0.008
**Depression**	Not experienced	(ref.)		
	Experienced	0.287	0.040	0.006
**Environmental factors**				
**Number of convenience stores per 1000 persons**		−0.257	0.225	0.371

^1^ S.E.: Standard Error; ^2^ ref.: reference group.

**Table 4:** Results of Logistic Regression Analysis for the Effects of Eating Experience at Convenience Stores on Good Self-Rated Health.

Independent Variables		OR ^1^	(95% CI ^2^)
**Gender**	Boys	1	
	Girls	0.708	(0.659–0.762)
**School**	Middle school	1	
	High school	0.740	(0.689–0.795)
**Father’s education**	≤High school	1	
	≥College	1.073	(0.976–1.180)
**Mother’s education**	≤High school	1	
	≥College	0.980	(0.891–1.077)
**Economic Status**	Upper	1.716	(1.542–1.910)
	Middle	1.273	(1.150–1.409)
	Lower	1	
**Living with family members**	Yes	1	
	No	0.946	(0.757–1.188)
**Vegetable intake**	<1 time a day	1	
	≥1 time a day	1.303	(1.211–1.402)
**Fruits intake**	<1 time a day	1	
	≥1 time a day	1.177	(1.080–1.283)
**Fast food intake**	<3 times a week	1	
	≥3 times a week	0.925	(0.849–1.006)
**Soda drinking**	<3 times a week	1	
	≥3 times a week	0.904	(0.835–0.979)
**Highly caffeinated beverage drinking**	<3 times a week	1	
	≥3 times a week	0.852	(0.766–0.947)
**Sugar-sweetened beverage drinking**	<3 times a week	1	
	≥3 times a week	1.088	(1.010–1.171)
**Alcohol drinking**	<1 time a month	1	
	≥1 time a month	1.012	(0.911–1.124)
**Smoking**	<1 cigarette a day	1	
	≥1 cigarette a day	0.914	(0.771–1.085)
**Sedentary time at weekend leisure time**	<4 h	1	
	≥4 h	0.865	(0.807–0.927)
**Sleep duration**	Not enough	0.643	(0.584–0.708)
	Enough	1	
**Stress perception**	Seldom	1	
	Often	0.445	(0.414–0.479)
**Depression**	Not experienced	1	
	Experienced	0.706	(0.653–0.762)
**Substituting a meal at convenience store**	<3 times a week	1	
	≥3 times a week	0.810	(0.750–0.875)

^1^ OR: Odds Ratio; ^2^ CI: Confidence Interval.
